# Enablers and inhibitors of exclusive breastfeeding: perspectives from mothers and health workers in Accra, Ghana

**DOI:** 10.1186/s13006-022-00462-z

**Published:** 2022-03-21

**Authors:** Martin Wiredu Agyekum, Samuel N. A. Codjoe, Fidelia A. A. Dake, Mumuni Abu

**Affiliations:** 1 Institute for Educational Research and Innovation Studies, University of Education, Winneba, P. O. Box 25, Winneba, Ghana; 2 Regional Institute for Population Studies, University of Ghana, P. O. Box LG 96, Legon-Accra, Ghana

**Keywords:** Exclusive breastfeeding, Decision making, Discontinuation, Enablers and inhibitors, Ghana

## Abstract

**Background:**

Despite the health and economic benefits of exclusive breastfeeding, there is evidence of a decline globally and in Ghana. Previous studies addressing this problem are mostly quantitative with only a few of such studies using qualitative or mixed methods to examine the predictors, benefits, ways of improving and managing exclusive breastfeeding, and the challenges associated with exclusive breastfeeding from the perspective of exclusive and nonexclusive breastfeeding mothers, and health workers. This study employs the health belief model to examine the experiences of mothers and health workers regarding exclusive breastfeeding to fill this gap in the literature.

**Methods:**

A cross-sectional qualitative study involving in-depth interviews was conducted among health workers and mothers attending child welfare clinic at two polyclinics in Madina, Accra-Ghana in 2019. Purposive sampling was used to select health facilities and participants for the study. Twenty participants comprising ten exclusive breastfeeding mothers, six non-exclusive breastfeeding mothers and four health workers were interviewed for the study. The data were analyzed based on emerging themes from inductive and deductive coding.

**Results:**

The decision to practice exclusive breastfeeding was based on mothers’ work, advertisement on exclusive breastfeeding and education on breastfeeding provided by health workers. Insufficient flow of breast milk, pressure from family and friends, and insufficient breast milk for infants were among the reasons for discontinuing exclusive breastfeeding. The factors that help improve exclusive breastfeeding include eating healthy food and breastfeeding on demand, while counselling and monitoring, restricting advertisement on infant formula and granting maternity leave for breastfeeding mothers were identified as factors that can  facilitate the practice of exclusive breastfeeding.

**Conclusion:**

Different levels of experience affect and shape exclusive breastfeeding practice in Ghana. The decision to practice exclusive breastfeeding, as well as the challenges and strategies employed in managing exclusive breastfeeding, emanates from mothers’ personal experiences and interactions with institutional factors. In view of this, there should be counselling on the management of challenges associated with exclusive breastfeeding and provision of accurate information on exclusive breastfeeding to enable mothers practice exclusive breastfeeding.

**Supplementary Information:**

The online version contains supplementary material available at 10.1186/s13006-022-00462-z.

## Background

Exclusive breastfeeding (EBF) is a public health intervention that provides optimal feeding as well as economic, social and health benefits for the reduction of morbidity and mortality among infants and mothers [1, 2]. Globally, empirical evidence shows that the prevalence of initiation of exclusive breastfeeding initiation is very high, but few mothers continue exclusive breastfeeding up to six months [3]. Early discontinuation of exclusive breastfeeding could be attributed to challenges of EBF and mothers’ inability to manage these challenges [4–9]. While on the one hand, factors such as insufficient milk production, lack of support for mothers, low confidence of mothers and pressure from family and friends have been identified as barriers to the practice of exclusive breastfeeding and thereby contributing to the low prevalence in Ghana and other sub-Saharan countries [10–13], on the other hand, other factors such as support for mothers, the flow of breast milk, benefits of exclusive breastfeeding, demand breastfeeding and interaction between mothers and health workers have been noted to contribute to the practice of exclusive breastfeeding [10–12].

The theoretical underpinnings of this study hinges on the Health Belief Model (HBM), on the premise that the decision of mothers to practice EBF depends on their knowledge of exclusive breastfeeding, the perceived benefits of exclusive breastfeeding, the perceived threats to exclusive breastfeeding and the cues to action for breastfeeding [14, 15]. This means that, if the perceived benefit of exclusive breastfeeding is greater than the perceived threat, a mother is likely to practice exclusive breastfeeding while the reverse may be true. However, there are cues to action that could facilitate exclusive breastfeeding. For example, strong support systems from family members, friends, and health workers could facilitate the practice of EBF and this could help to reduce discontinuation and the risk of non-exclusive breastfeeding.

Additionally, the practice of exclusive breastfeeding depends on the understanding and the ability of mothers to overcome the associated challenges. This, includes creating an enabling environment at home, at work and in health facilities that support mothers to practice exclusive breastfeeding [1, 4, 10, 16]. Evidence from research shows that the experience of mothers is shaped by their interaction with other women most especially health workers [5]. Health workers play a significant role in women’s decision-making process regarding EBF as well as in the management of exclusive breastfeeding. Nieuwoudt and Manderson [5] argue that mothers rely mostly on the advice of health workers to decide to exclusively breastfeed and manage EBF. Hence, providing information to mothers on the challenges and management of EBF could lead to the continuation of EBF for the recommended six months. However, many mothers are not provided information on EBF and they do not have knowledge on the management of exclusive breastfeeding, thus inhibiting their ability to practice EBF. Consistent provision of information on the management and improvement of exclusive breastfeeding could thus help mothers understand exclusive breastfeeding better and enable them to overcome the challenges associated with exclusive breastfeeding [17].

In Ghana, according to the 2014 Ghana Demographic and Health Survey, there has been a decline in the prevalence of exclusive breastfeeding as measured using the 24-h recall method. The prevalence of exclusive breastfeeding has been observed to have declined from 63% in 2008 to 52% in 2014 [18]. Among several factors, conditions in the home and work environments, maternal and paternal characteristics and societal influence contribute to this decline in EBF [10, 19, 20]. For example, mothers who work in formal settings are unable to practice EBF due to an unfriendly breastfeeding environment at the workplace [10]. Hence, there have been discussions on extending the current three (3) months maternity leave policy in Ghana to six (6) months to help especially working mothers’ practice exclusive breastfeeding. Such policy revisions are critical because of the benefits of EBF for the health of infants, mothers and the nation at large. Revising this policy will help women to spend more time with their children after delivery and offer them the opportunity to practice exclusive breastfeeding.

Several studies have been conducted on exclusive breastfeeding, however, existing studies in Ghana and sub-Saharan Africa are mostly quantitative with few studies using qualitative or mixed methods [10, 12, 19, 21–23]. There is thus a paucity of qualitative studies that focus on challenges, benefits and cultural factors affecting exclusive breastfeeding [11, 13, 24]. Again, only a few studies have examined the experiences of exclusive and non-exclusive breastfeeding mothers, and health workers to understand the complexities and nuances surrounding decision making, challenges and management of the challenges associated with practicing EBF in Ghana. Furthermore, previous studies have focused largely on mothers but health workers are the first point of contact with mothers especially at birth and health workers serve as advocates for exclusive breastfeeding [5]. It is therefore important to study the perspectives of health workers on exclusive breastfeeding, as the information provided by health workers could offer the needed support for mothers to overcome the challenges associated with exclusive breastfeeding [7].

Against the foregoing, the objective of this study is to examine experiences of practicing exclusive breastfeeding, from the perspective of exclusive and non-exclusive breastfeeding mothers. The study also explores the perspectives of health workers who interact often with peripartum women.

## Methods

### Study design

This was study designed as a cross-sectional qualitative study. In-depth interviews were conducted among mothers in their reproductive age (15–49 years) and health workers in the La Nkwantanang Municipality (Madina) in Accra, Ghana. This study was embedded within a cross-sectional mixed-methods study under the “Willows Impact Evaluation” project conducted in Ghana and implemented by the Regional Institute for Population Studies at the University of Ghana. The larger study explored reproductive behaviours of contraceptive use among urban poor women along selected coastal towns (Osu klottey, La, Teshie and Nungua) and inland areas (La Nkwantanang, Abogba and Old Ashongman) in Accra, Ghana [25].

### Setting

The study was conducted in two health facilities at Madina-Accra, Ghana. Madina is the municipal capital of the La-Nkwantanang Municipal Assembly. It is located in the northern part of the Greater Accra Region and covers 70.887 square kilometres. The Municipality shares boundaries in the west with Ga East Municipal, the east with Adenta Municipal, the south with Accra Metropolitan Assembly and the north with Akuapim South District [26]. According to the 2010 Ghana Population and Housing Census report, the population of the La-Nkwantanang Municipality is youthful (38.7%) with a small (5.0%) proportion of older adults [26]. The indigenous people of the municipality are Ga-Dangbes. However, most of the current residents are migrants with the majority of in-migrants coming from the Eastern and Volta regions. The main economic activities are commerce, agriculture, services and manufacturing. The Madina market serves as the main trading centre in the municipality [26]. In terms of health facilities, Madina has both private and public health facilities. The public health facilities include two polyclinics (Kekeli Polyclinic and Rawlings Circle Polyclinic), health centres, and Community-Based Health Planning and Service (CHPS) compounds.

### Selection of participants

Purposive sampling was used to select the health facilities and the participants for this study. The two polyclinics in the La-Nkwatanang Municipality, namely; Kekeli Polyclinic and Rawlings Circle Polyclinic were purposively selected based on the fact that they focus more on providing maternal and child health services to a wide population in Madina and its neighboring environment. Breastfeeding mothers attending child welfare clinic in the two polyclinics were purposively selected for the study (with the help of health workers) based on the following criteria;

- The mother must be within the reproductive age of 15–49 years,
- Must have an infant who is less than 6 months old,
- Must be practicing exclusive breastfeeding (Feeding infants aged 0 to 5 months with only breast milk and no other liquids, solids or water except for oral rehydration solution, drops) or not practicing exclusive breastfeeding (mothers who fed their infants with breast milk and or any other food; classified as non-exclusive breastfeeding)

Health workers were also purposively selected for in-depth interviews based on the following criteria;

- Should be a permanent staff of the health facility.
- Provides services to pregnant women and breastfeeding mothers.

In all, twenty (20) participants were interviewed for the study. This includes ten mothers who were practicing exclusive breastfeeding, six mothers who were not practicing exclusive breastfeeding and four health workers. In each health facility, five mothers who were exclusively breastfeeding, three mothers who were not exclusively breastfeeding and two health workers were interviewed.

### Data collection

Data collection for the study was carried out in June 2019 using a semi-structured interview guide that was developed and pilot-tested for the study. The interview guides consisted of sub-section on the demographic characters of mothers, child’s characteristics, decision to exclusively breastfeed or not, as well as the management, challenges and benefits of exclusive breastfeeding (An English version of the questionnaire has been uploaded as Additional file 1, Additional file 2 and Additional file 3). The participants were selected for interview through the following process. Upon entering the hospitals, permission was sought from the management to use the facility for the study. Permission was then sought from the participants (mothers and health workers) to be included in the study. The purpose of the study including the general objectives, benefits, and risk of taking part in the study were explained to the participants. After that, the respondent, if literate, signed an informed consent form and, if they could not read or write, provided a thumbprint or verbal consent to participate in the study. Participants were also informed about strict confidentiality and anonymity of their responses. Lastly, they were informed about their right to stop participating in the study at any point if they desired to do so. The health workers were interviewed in an office while the interviews with mothers were conducted at a quiet location within the health facility without interference or the presence of other people. In recruiting respondents, Not all the mothers who were approached agreed to be part of the study. Those who refused to be interviewed cited work and husband approval as their reasons for not being part of the study. Only those who agreed to be part of the study were interviewed. All the interviews were carried out by the lead author. The interviews were conducted in a preferred language of the participants, mainly English and Twi. The interviews were audio-recorded with the permission of the participants.

### Data analysis

The data were analyzed thematically using Atlas.ti. The thematic analysis approach involves searching across a range of text or transcripts to find a repeated pattern of meaning and organising these into various levels of themes such as basic, organising and global themes [27]. According to Braun and Clarke [27] there are six steps in the analyses of qualitative data and these are; familiarizing yourself with the data, generating initial codes, searching for themes, reviewing themes, defining and naming themes and producing a report. The initial stage involves reading through the transcripts repeatedly to gain familiarity with the text as well as noting initial ideas. This was followed by assigning codes to the appropriate segment of the transcripts. The assigned codes are organized to generate cross-cutting themes from all the transcripts. At this stage, different codes from the transcripts are sorted into potential themes, as all relevant codes are collated within the identified themes [27]. The various codes are searched, reviewed and named for the production of a report.

In analyzing the data, all the authors read through the transcripts to gain familiarity with the text. Inductive coding and deductive coding approaches were used in the data analysis. The first author read through all the transcripts to identify the deductive codes and themes. The co-authors provided feedback on the identified codes and themes after each of them had gone through the transcripts. The lead author and one co-author coded all the responses and generated the various themes. The other two co-authors validated the codes and the various themes. Thematic networks maps were constructed to show the relationship between the various themes at the three levels: basic, organising and global themes. Pseudo names were used in reporting quotes from the transcripts.

### Ethical approval

The present qualitative study was conducted as part of the Willows Impact Evaluation Study. Ethical approval for the Willows International Evaluation Project was granted by the Ethics Review Committee of Ghana Health Service (GHS-ERC #005/08/2017) and the University of Ghana Ethics Committee for the Humanities (ERC #020/17–18). The ethical clearance was further used to obtain administrative permission from the two polyclinics and from the study participants, i.e. mothers and health workers.

## Results

### Characteristics of study participants

As shown in Table 1, ten of the sixteen mothers interviewed practiced exclusive breastfeeding while the remaining six were not practicing exclusive breastfeeding. The age range for the mothers and health workers was 25 to 40 years. About 7 of the mothers and health workers had Junior High School level of education and a total of sixteen (16) of the mothers and health workers were Christians. With regards to marital status, 16 of the participants were married. The health workers who were interviewed as part of this study included a community health nurse, a nutritionist, a midwife and a registered community nurse.

**Table 1:** Characteristics of exclusive and non-exclusive mothers and health workers interviewed for the study

Characteristics	Exclusive breastfeeding mothers (*N* = 10)	Non-exclusive breastfeeding mothers (*N* = 6)	Health workers (*N* = 4)	Total Number of Participants (*N* = 20)
**Age of mother**				
25–30	3	4	1	8
31–35	5	2	2	9
36–40	2	0	1	3
**Level of education of mother**				
Primary	1	0	0	1
JHS	6	1	0	7
SHS	1	4	0	5
Tertiary	2	1	4	7
**Ethnicity**				
Akan	1	3	3	7
Dagomba	2	1	0	3
Gonja	2	1	0	3
Ewe	4	0	1	5
Ga	1	0	0	1
Other (Nigerian)	0	1	0	1
**Religious affiliation**				
Muslim	3	1	0	4
Christian	7	5	4	16
**Marital Status**				
Never married	2	1	1	4
Married	8	5	3	16
**Employment status**				
Employed	8	5	4	17
Unemployed	2	1	1	3
**Category of health worker**				
Nutritionist	-	-	1	1
Midwife	-	-	1	1
Community Health Nurse	-	-	1	1
Registered Community Nurse	-	-	1	1

### Thematic analyses

Five major organising themes that are related to the global theme (exclusive breastfeeding practice) were identified. These are (i) decision to practice exclusive breastfeeding, (ii) challenges of exclusive breastfeeding, (iii) reasons for discontinuation of EBF, (iv) management of EBF and (v) improvement of exclusive breastfeeding.

### Decision to practice exclusive breastfeeding

**Fig. 1:**
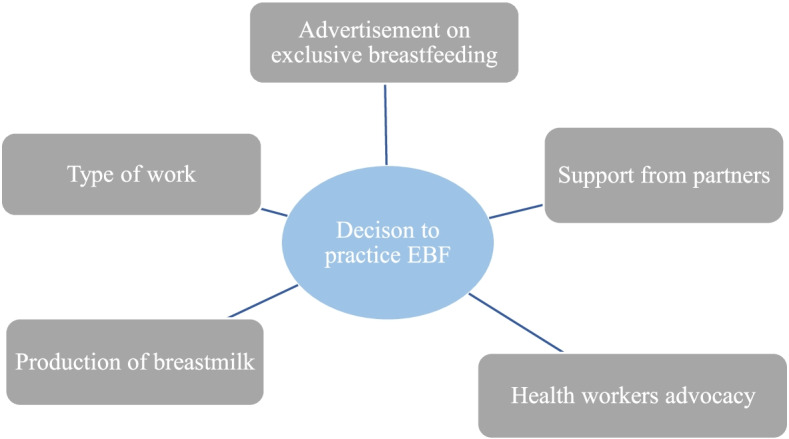
Decision to practice exclusive breastfeeding

The results reveal that the decision of mothers to practice exclusive breastfeeding was based on advertisement, type of work, the ability of mothers to produce enough breast milk, health workers advocacy and support from partners (Fig. 1).

### Advertisement for exclusive breastfeeding

Advertisement was identified as an important factor in the decision regarding exclusive breastfeeding as it informs and provides mothers with the needed information on the importance of exclusive breastfeeding thereby influencing their decision to practice EBF. This was highlighted by Abena, an exclusive breastfeeding mother;

*“I have seen an advertisement that says that if you give the baby only breast milk, it develops her brains. That is why I planned to give her only breast milk”* (Exclusive breastfeeding mother, 36 years).

### Type of work

Type of work facilitates or hinders the decision of mothers to practice exclusive breastfeeding. Mothers working in formal settings are unable to practice exclusive breastfeeding for long compared to mothers working in informal settings. This is due to unfavourable working conditions in the formal sectors such as separation of the child from the mother and lack of private room/space for breastfeeding. Most of these mothers leave their children at home when they resume work from their three months maternity leave, thus making it difficult for them to breastfeed on demand. However, mothers working in informal settings are always with their children and they are able to breastfeed and practice exclusive breastfeeding much longer. This, therefore, influences the mother’s decision on exclusive breastfeeding. The quote below from Edna highlights this point.

*“Because of the nature of my work (trading), I think that’s a factor which made me practice exclusive breastfeeding. Those in the government sector such as banks, if you want to practice exclusive breastfeeding, it would be quite difficult. Even if you pump breast milk and place it in the fridge for the child, I don’t think it would be healthy for the child. The nature of my work also helps me practice exclusive breastfeeding because my child is with me always and I can take her everywhere, I go”* (Exclusive breastfeeding mother, 30 years).

### Production of breast milk

The ability of mothers to produce enough breast milk also influences their decision to practice exclusive breastfeeding. For example, Agnes reported that;

*“I have a lot of breast milk so I do not see why I should add any other thing when the breast milk is there. I always give and she is always satisfied after taking it. So, I don’t have to add anything”* (Exclusive breastfeeding mother, 40 years).

Additionally, one of the health workers, a midwife, indicated that.

*“You can easily access breast milk when the baby is crying, it is available, and you do not prepare or have to go to the kitchen. It is available and brings love. That also influence their decision to practice exclusive breastfeeding”* (Midwife, 40 years).

### Health workers advocacy

Health advocacy including talks on exclusive breastfeeding by health workers at the health facilities during antenatal and postnatal clinics played a critical role in mothers’ decision-making. For instance, some mothers mentioned that during post-natal and antenatal clinics, health workers taught them how to breastfeed their babies. Sometimes they use a doll to demonstrate how to hold the baby and breastfeed. They are also taken through nutrition lessons including exclusive breastfeeding management, benefits, and challenges. This helped them to understand exclusive breastfeeding practice and thereby influence their decision to practice exclusive breastfeeding. In addition, the health workers indicated that when mothers understand the education received at the health facilities on exclusive breastfeeding, they are encouraged to practice exclusive breastfeeding. One of the health workers mentioned that;

*“We normally explain exclusive breastfeeding to them for their understanding. If they understand the benefits of breastfeeding, then there is a need for them to start”* (Midwife, 40 years).

Similarly, a 34-year-old mother, Esther, reported that.

*“Yes, they really teach us how to breastfeed. They sometimes bring a doll and teach us how we should hold the breast, how you should feed the baby, the kind of food you can give it and how to keep yourself neat. This, therefore, influenced my decision to practice exclusive breastfeeding”* (Exclusive breastfeeding mother, 34 years).

### Partner support

Another factor that was found to promote the decision on exclusive breastfeeding was partner support. Partner support is very important in women’s decision to practice exclusive breastfeeding. All exclusive breastfeeding mothers indicated that they had support from their partners regarding their decision to practice exclusive breastfeeding. Some mentioned that their partners wanted them to practice exclusive breastfeeding for a year. For example, Mavis, an exclusive breastfeeding mother reported that.

“I did exclusive breastfeeding because my husband understands and supported me to practice exclusive breastfeeding” (Exclusive breastfeeding mother, 30 years)

Similarly, Sandra, a breastfeeding mother indicated that her husband provided the needed support to ensure that she gives only breast milk to the infant. She mentioned  that;

*“I discuss with my husband to give her only breast milk... he agreed and wouldn’t even allow me to give her any other food. He provided security to ensure that I don’t give the child any food apart from breastmilk”* (Exclusive breastfeeding mother, 35 years).

### Challenges of exclusive breastfeeding

**Fig. 2:**
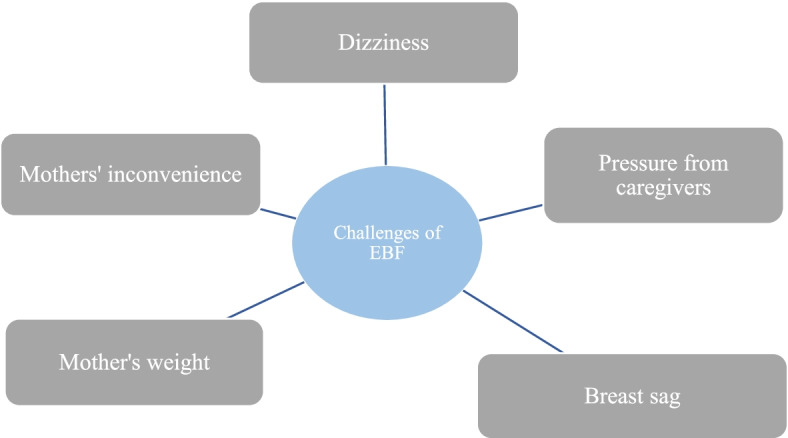
Challenges of exclusive breastfeeding

Five sub-themes including, dizziness among mothers, inconvenience of mothers, mothers’ weight, breast sag and pressure from caregivers were identified by health workers and mothers as challenges of exclusive breastfeeding (Fig. 2).

### Dizziness

Exclusive breastfeeding mothers indicated that after breastfeeding, they become dizzy and are unable to perform any other activity. This makes them restless for some time before gaining strength to perform normal activities. One participant, Mary, indicated that;

*“There are times when you breastfeed your baby, you will feel dizzy and you will have to lie down because you will not be able to do anything”* (Exclusive breastfeeding mother, 32 years).

### Mothers’ inconvenience

Another challenge mentioned by exclusive breastfeeding mothers was inconvenience. The mothers indicated that because they breastfeed on demand, the child is always with them wherever they go and this sometimes inconveniences or limits their movement. For instance, Bernice reported that.

*“Because of her (the child) feeding, you would have to take her everywhere you go. You cannot tell somebody to take care of your child”* (Exclusive breastfeeding mother, 30 years).

### Weight of mother

Additionally, some mothers indicated that they put on weight because they eat more food to enable them produce breast milk. Some of the mothers were uncomfortable with this situation as their appearance changes. For example, Edna reported that;

*“Because of exclusive breastfeeding you put on weight even when you do not want to, you have to eat so the baby can also benefit. Due to this, you put on weight by so doing”* (Exclusive breastfeeding mother, 30 years).

### Breast sag

There was a perception by the mothers that practicing exclusive breastfeeding is associated with breast sag. As a result of this, they shorten the practice of exclusive breastfeeding. However, a midwife explained that this is a misconception and ignorance on the part of the mothers. The midwife emphasized that breast sag is not as a result of exclusive breastfeeding or prolonged breastfeeding. She reiterated,

*“…. that is ignorance because whether you breastfeed or not your breast will sag. They do not know because if they knew they wouldn’t say that. Because if you have a small breast that cannot come down, it will not come down… It is the weight that sags the breast. What about those who haven’t delivered, and their breasts are sagging? Will the men that suck their breast sag their breast or not? I think those who are educated are the problem”* (Midwife, 40 years).

### Pressure from caregivers

Also, both health workers and mothers mentioned that pressure from family and friends, mostly caregivers, are challenges associated with exclusive breastfeeding. A health worker indicated that caregivers, mostly mothers-in-law who are not in support of exclusive breastfeeding, sometimes give water and food to the children without the knowledge of the child’s mother. This sometimes leads to the child developing infectious diseases because of the contamination of food that they feed the children. One of the health workers attested to this and she indicated that;

*“Our challenge is the caregivers; the caregivers are mothers-in-law who are their own mothers. They are old and did not practice exclusive breastfeeding, so they don’t want to believe that what we are saying is right. At times they even hide to give water to the child. When they see that the mother insists that they do not have to give water, during bathing they will give water to the child”* (Midwife, 40 years).

Similarly, one of the mothers mentioned that she was pressurised by her friends to give her baby food.

“*My friends told me he will not have an appetite for food when he grows up and I told them I will start giving him beans, maize and rice when he is six months. I believe he will have an appetite for food when he turns 6 months”* (Exclusive breastfeeding mother, 28 years).

Aside from mothers-in-law, some friends discouraged mothers from practicing exclusive breastfeeding. They explain to mothers that exclusively breastfed children do not eat well after six months of exclusive breastfeeding. One of the mothers reported that;

*“Some of my friends told me to give him food, while others said no, but I had decided to practice exclusive breastfeeding. When they ask me to give the baby food, I only said yes knowing that I would not do it”* (Exclusive breastfeeding mother, 30 years).

### Reasons to discontinue exclusive breastfeeding

**Fig. 3:**
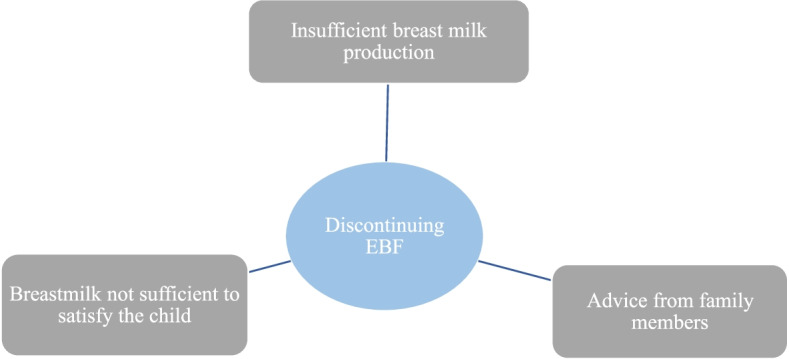
Factors contributing to discontinuation of exclusive breastfeeding

Some mothers started with exclusive breastfeeding but discontinued because of advice from family members, insufficient breast milk for the child and insufficient flow of breast milk (Fig. 3).

### Insufficient breast milk production

Insufficient breast milk production is when the mother may not produce enough breast milk to feed the infant. The lack of production of breast milk could lead to a situation where the mother does not exclusively breastfeed at all or discontinues exclusive breastfeeding early. Some mothers mentioned that they wanted to practice exclusive breastfeeding but when they realised that they were not producing enough breast milk, they introduced other supplementary foods to the child. Akua, for example, reported that;

*“I planned practicing exclusive breastfeeding when I give birth. There are some people who do not have breast milk naturally, when I gave birth the breast milk wasn’t flowing so we added other foods”* (Non-Exclusive breastfeeding mother, 27 years)

### Breast milk not sufficient to satisfy the child

According to the account of the respondents, the situation where breast milk is not sufficient to satisfy the child arises where the mother produces enough breastmilk but perceives that breast milk alone is not enough or sufficient to satisfy the child, hence there is the need to give the child other supplementary foods. Some of the respondents indicated that breast milk was not enough for their children. They attributed this to the fact that their children were not able to sleep well at night and they had to introduce other foods such as coconut water and SMA to enable them to sleep. One mother indicated that;

***“****When I gave birth, I was giving her only breast milk, but she was not sleeping, she kept crying at night. So, I thought it was because of what she ate, so I first gave her coconut water and after drinking it she slept throughout the night. I could not give her coconut water all the time because the peel of the coconut might be in the water... So, I gave her SMA for a month and then I stopped”* (Non-Exclusive breastfeeding mother, 26 years).

Also, a health worker expressed that most mothers add food supplements because they feel that breast milk is not enough. She reiterated that;

*“Some mothers say breast milk is not enough for their children. All the time when they are eating, their children also feels like eating. They think it’s not enough, so they have to add some food supplements to it”* (Nutritionist, 31 years).

### Advice from family members

Advice from family members’ especially biological mothers of breastfeeding mothers and mothers-in-law contribute to the discontinuation of exclusive breastfeeding. A mother expressed that she wanted to practice exclusive breastfeeding up to six months, but her mother advised her to give water and food to the child, and this discontinued the practice of exclusive breastfeeding. She indicated that;

*“He will be six months next month so I started the water last week because my mum said there are some things if he doesn’t get used to , he wouldn’t like  them. He should be taking water, to get him going, so if he is not used to it now, he is now going to start when he is six months, and he will get used to it in the eighth or ninth month. So I should start giving him small, so he knows this is water”* (Non-Exclusive breastfeeding mother, 26 years).

Aside mothers who discouraged their daughters from practicing EBF, mothers-in-law also discouraged breastfeeding mothers to discontinue EBF. Ernestina reported that.

*“My mother-in-law was not in support. Anytime the baby cries she thinks the baby is hungry, so she will ask me to give the baby porridge or something else”* (Non-Exclusive breastfeeding mother, 27 years).

### Management of exclusive breastfeeding

**Fig. 4:**
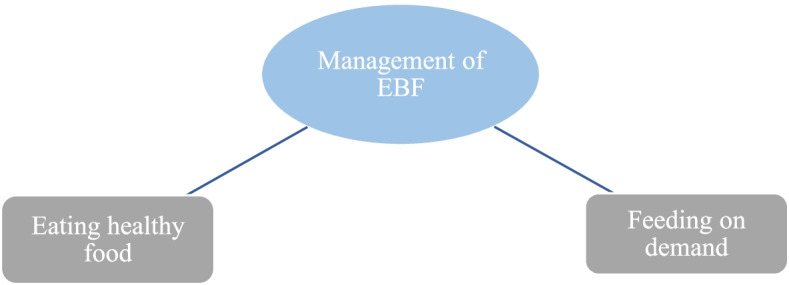
Management of exclusive breastfeeding

Despite the challenges associated with exclusive breastfeeding, there appeared to be ways exclusive breastfeeding is managed by mothers. According to Fig. 4, these include eating healthy food and feeding on demand.

### Eating healthy food

Eating healthy foods was identified as one of the ways of managing exclusive breastfeeding practice. Majority of the exclusive breastfeeding mothers indicated that there are specific healthy meals such as groundnut soup, palm nut soup, mashed kenkey, and fruits that help in the production of breast milk. Mothers eat these foods regularly to enable them to produce enough breast milk. Abena a 36 years old exclusive breastfeeding mother explained that;

*“You must eat well. You must drink more soup-like palm nut soup and groundnut soup. That will give you more milk. Also, mashed Fante kenkey with groundnut also helps to produce more milk for the baby”* (Exclusive breastfeeding mother, 36 years).

Another participant, Esther also indicated that;

*“I only have to eat healthy foods. I must eat to my satisfaction because they normally say the breast milk is dependent on the food you eat. They also say if the mother eats healthy foods, she will produce healthy milk for the baby. I can prepare “tuo zaafi with ayoyo” (maize dish) in the morning. We drink “agushi soup” and I eat fruits. After every meal, I eat fruits”* (Exclusive breastfeeding mother, 34 years).

### Feeding on demand

Also, breastfeeding on demand was identified as one of the ways of managing exclusive breastfeeding. Exclusive breastfeeding mothers mentioned that they keep an eye on their children in order to breastfeed when needed. Mothers breastfeed at regular intervals and do not wait for the child to cry for food. Some mothers indicated that in their absence, they express breast milk for caregivers to breastfeed their children. For instance, Bertha indicated that.

*“You must keep an eye on him so that you can breastfeed him regularly. You should not wait for a long period before you breastfeed him. You should be feeding him regularly so that he will not cry and disturb. He doesn’t cry and disturb me”* (Exclusive breastfeeding mother, 28 years).

Similarly, time management is very important in exclusive breastfeeding. Some participants expressed that having time for the baby enables them to monitor the wellbeing of the child as well as breastfeeding well. This includes prioritising the baby over any other thing as Abena indicated;

*“I have to make time for the baby. If I don’t have time for the baby, he will worry me, and I will be tempted to add other foods when he is not 6 months old. I have to make time for the baby and prioritize the baby over my work”* (Exclusive breastfeeding mother, 36 years).

### Improvement in exclusive breastfeeding

**Fig. 5:**
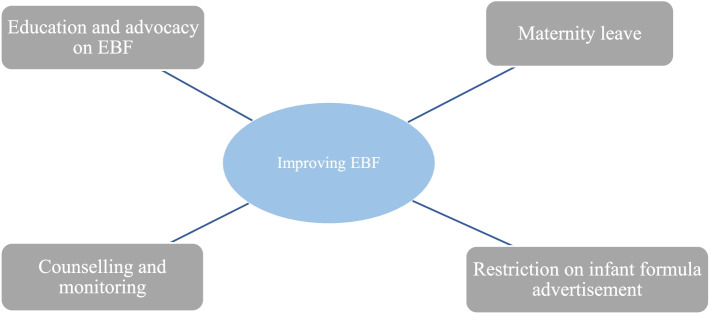
Improving exclusive breastfeeding

Health workers identified several strategies through which exclusive breastfeeding could be improved. This includes education on EBF, counselling and monitoring mothers, restricting advertisement on infant formula and maternity leave (Fig. 5).

### Education on EBF

Narrations of the health workers showed that education on exclusive breastfeeding at antenatal and postnatal clinics, provides mothers with the necessary knowledge about EBF and creates awareness on exclusive breastfeeding practice. The health workers also mentioned that, education on exclusive breastfeeding should not target only mothers but others including grandmothers, partners, and in-laws. One of the health workers reported that;

*“It can be improved through health education during antenatal services, postnatal services, child welfare clinic even including those at the OPDs… when we give talks, they are not excluded. It’s not only pregnant women that we must talk to, but we also have men as partners who are also involved because it’is not only mothers. Sometimes those at the OPDs like grandmothers, caregivers are also part. So, when we give talks like that, it is also extend to them”* (Nutritionist, 31 years).

Another health worker supported the intensification of education and mentioned that.

“*We must intensify our education at our antenatal clinics, child welfare clinics, and the communities. It is more of the education”* (Registered Community Nurse, 35 years).

Also, the health workers indicated that sometimes mothers find it easy to understand their own peers than the health professionals. In such situations, the health workers use mothers who have been successful in practicing exclusive breastfeeding to talk to their fellow mothers about misconceptions and benefits of exclusive breastfeeding practice. This motivates and encourages others to practice exclusive breastfeeding. A registered community health nurse indicated that;

*“They tell them the baby will be thirsty if you don’t give him water, but we explain to them that breast milk contains water and they understand it. But if you want them to understand, then you use a colleague who has done exclusive breastfeeding and has been successful like I said. That is the best way to teach a mother”* (35 years, Registered Community Nurse).

### Counselling and monitoring

Furthermore, counselling and monitoring of women were mentioned as one of the strategies for improving exclusive breastfeeding. A health worker indicated that due to the misconceptions about exclusive breastfeeding, most mothers were put on counselling programs and then monitored. Through this, mothers have understood the benefits, misconceptions and challenges associated with exclusive breastfeeding and they are now encouraged to practice exclusive breastfeeding. A health worker expressed that counselling has helped women to understand the benefit of exclusive breastfeeding and this should continue;

*“With much counselling and monitoring, they are now understanding the main reason(s) why you should exclusively breastfeed your baby”* (Community Health Nurse, 25 years).

### Controlling advertisement on infant formula

Controlling advertisement on infant formula feeding was identified in the narrative to enhance exclusive breastfeeding. Restrictions on advertisement of infant formula could help to reduce the patronage of these foods, which consequently, could help to improve the practice of exclusive breastfeeding. A community health worker mentioned that;

*“If the advertisement of infant formula food can go down, most people will love to breastfeed because if you don’t know of a formula that you can give to your baby, you will definitely breastfeed because you want your baby to live”* (Community Health Nurse, 25 years).

### Maternity leave

In addition, the health workers mentioned that working mothers do not get to have enough time to practice exclusive breastfeeding due to the short duration of maternity leave. The working environment does not allow mothers to go to work with their children to breastfeed on demand. This therefore limits their ability to exclusively breastfeed for six months. For instance, a midwife gave an account of a colleague nurse who closed at work early to take care of the child at home.

*“We said mothers should take leave up to 6 months so they can breastfeed their babies. Like the lady (nurse) who closed from work to go home and breastfeed. The child is 3 months so if she has maternity leave for 6 months she would not have come to work, she would have stayed at home to breastfeed, but the challenges are there. I think that will be the only way that can help to make exclusive breastfeeding effective. Mothers should be with their babies for 6 months. The sixth month you introduce the solid foods then you start work”* (Midwife, 40 years).

### Enablers and inhibitors of EBF

**Fig. 6:**
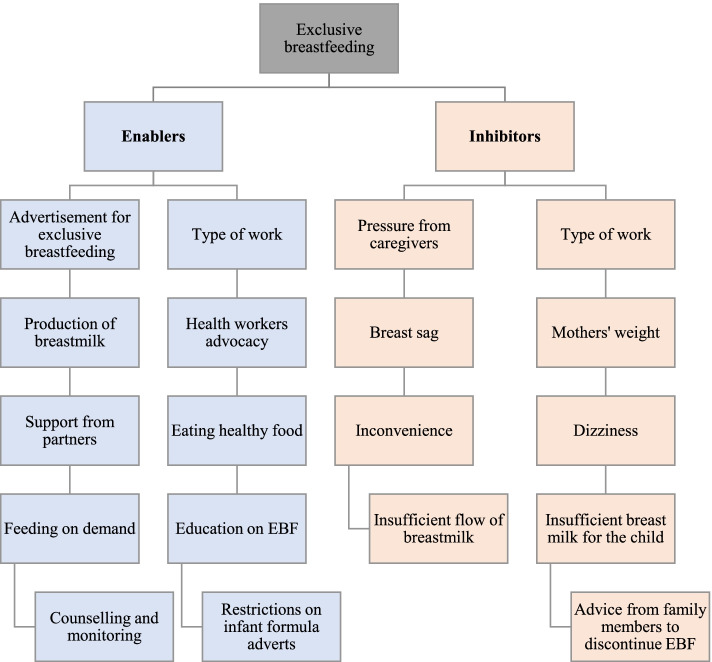
Framework showing the enablers and inhibitors of EBF in Ghana

Figure 6 provides a summary of the results of the study. It captures the enablers and inhibitors of exclusive breastfeeding practice. The enabling factors are presented first, on the left side in the diagram while the inhibiting factors are also presented on the right side of the diagram. Among some enablers of exclusive breastfeeding are advertisement for exclusive breastfeeding, type of work, production of breast milk, health workers advocacy, support from partners, eating healthy food, feeding on demand, education on exclusive breastfeeding, counselling and monitoring, maternity leave and controlling advertisement on infant formula. On the other hand, the factors that inhibit the practice of exclusive breastfeeding include the type of work, pressure from caregivers, breast sag, mothers’ weight, dizziness, mothers’ inconvenience, insufficient breast milk production, breast milk not being sufficient to satisfy the child and advice from family members.

## Discussion

Previous studies have highlighted the benefits, challenges and cultural practices affecting exclusive breastfeeding practice in Ghana and SSA [10, 13, 16, 28, 29]. However, there is scarce literature on the decision to practice EBF, reasons for discontinuation of EBF, management and ways of improving EBF. This study contributes to the exclusive breastfeeding debate by highlighting experiences of exclusive breastfeeding mothers, non-exclusive breastfeeding mothers and health workers in relation to factors that contribute to women’s decision to practice EBF, reasons for discontinuing EBF, challenges associated with EBF, management and ways of improving EBF. Additionally, by, using the health belief model, this study triangulates the experiences of mothers and health workers to understand the complexity of exclusive breastfeeding in Ghana. The results of the study suggest that there are individual, interpersonal, and organizational factors that explain the complexity of exclusive breastfeeding in Ghana. These factors are shaped by the experiences of mothers and their interaction with family members, friends, and other stakeholders including health workers.

The findings indicate that decision making on exclusive breastfeeding is influenced by multiple factors. From the results, the dominant themes among these factors are education by health workers and mothers’ work. The findings underscore the relevance of accurate information on exclusive breastfeeding and therefore places an emphasise on education by health workers. Previous studies have reported the influence of education by health professionals on mothers’ decision making regarding exclusive breastfeeding [11, 16, 30, 31]. In this study, the plausible reason could be that health workers communicate clearly to mothers by demonstrating how to breastfeed using objects such as dolls and other relevant materials. In addition, observations made by the lead author during data collection show that health talks are given almost every morning at child welfare clinics using simple English and local languages. These talks sometimes take the form of an interactive section where mothers are allowed to ask questions and health workers respond to the various questions. This probably increased mother’s self-confidence and offered them security in their decision to practice exclusive breastfeeding [31]. Such approaches could be leveraged as a way of reaching out to mothers with, exclusive breastfeeding programs designed to reach women through social media outlets such as television and radio.

The results also suggest that the work schedules of mothers play a significant role in their decision to practice exclusive breastfeeding. Informal work such as trading enables mothers to be in contact with their children always, unlike formal work which separates mothers from their children. Also, unfriendly working facilities in formal workplaces  limits the ability of mothers to continue exclusive breastfeeding after resuming work from their maternity leave. In Ghana, there are not enough enabling or supportive environments at the various work places to accommodate mothers and their babies [10]. There is lack of privacy for breastfeeding, and most mothers are not allowed to go to work with their babies. This limits the ability of mothers to continue practicing exclusive breastfeeding after going back to work and therefore affects the bonding between the mother and the child at the early formative stages. The findings, therefore, have implications for policies at the work place on exclusive breastfeeding, i.e. encouraging organisations to provide supportive working environment for breastfeeding mothers. Provision should also be made for mothers to take their children to work or there should be flexible working terms for breastfeeding mothers such as working from home if there is the need.

This study identified several challenges associated with exclusive breastfeeding and these challenges have implications for policy for the consideration of health practitioners. For example, there were complaints of dizziness after exclusive breastfeeding by mothers as they indicated that it makes them inactive for some time. Similar health challenges have been reported in Australia where exclusive breastfeeding mothers experience health problems after breastfeeding [32]. The implication is that when such challenges are not addressed, mothers may discontinue exclusive breastfeeding.

Other reasons for discontinuation of exclusive breastfeeding focused on insufficient production of breastmilk, pressure from family members and breastmilk not being sufficient to satisfy the child. Some participants expressed that naturally, they are unable to produce enough breast milk to feed their children. Insufficient breast milk production could be as a result of a medical or non-medical problem that disrupts the practice of exclusive breastfeeding. This underscores the relevance of counselling and education to identify the cause of insufficient flow of breast milk to help mothers in managing breastfeeding challenges.

Also, eating healthy food, and breastfeeding on demand were strategies adopted for managing exclusive breastfeeding. Breastfeeding mothers’ believe that eating such foods will help them to produce enough breastfeeding. Mothers indicated that they needed to eat healthy food in order for them to produce enough breast milk. These foods include mashed kenkey and soups. Consistent with other studies in Tanzaina, Mgongo et al., [24] reported that special food such as soup and kitawa (mashed banana with sour milk) are prepared for women to produce enough breastmilk. The probable reason could be that soup, mashed kenkey and sour milk have nutritional value which helps breastfeeding mothers to produce enough breast milk. It is therefore important to have nutritionists based at health facilities especially, in the rural areas to provide advice to mothers on the specific type of food to eat in order to produce breast milk.

The results of the study show that restrictions on advertisement is very vital and serve as a way of improving exclusive breastfeeding practice. This is because in Ghana there is an influx of advertisement on infant formula which restraints women from practicing exclusive breastfeeding. Women use these infant formulae feeds as a supplement or complement to breastfeed their infants [33–35]. But if there are restrictions on infant formula, mothers will have no alternative and will be motivated to practice exclusive breastfeeding. Moreover, health workers mentioned that there was the need to use women who have been successful in practicing exclusive breastfeeding to educate and create awareness on exclusive breastfeeding. This is because women tend to learn and understand the language of their own colleagues than health workers. Also, when women are allowed to share their experiences, other women understand their experiences better than the health workers explaining to them.

In terms of addressing challenges associated with exclusive breastfeeding, counselling and monitoring were identified as another strategy for improving exclusive breastfeeding. Health workers adopted this method in identifying challenges associated with exclusive breastfeeding and helping mothers to overcome these challenges. It was realized that, counselling and monitoring has an impact on improving exclusive breastfeeding in Ghana. It is therefore very important that breastfeeding counselling is performed during antenatal care sessions and after delivery during the mother's stay at the hospital. In addition, there should be follow up counselling at postnatal clinics and home visits to help improve exclusive breastfeeding practice.

Lastly, the short maternity leave of three months limits the ability of mothers working in formal settings to practice exclusive breastfeeding for six months. Several studies have reported that mothers working in formal environments discontinue exclusive breastfeeding when they resume work after the three months of maternity leave [10, 28, 33]. In view of this, there is the need for the Government of Ghana to revise the three months policy to six months to help mothers’ practice exclusive breastfeeding for six months.

The findings of this study support the position of Health Belief Model. The findings give indication that breastfeeding mothers have a favorable understanding of the perceived benefits of practicing exclusive breastfeeding as well as the dangers associated with non-exclusive breastfeeding practices (perceived severity). This, therefore, enforces the desire of mothers to practice exclusive breastfeeding. However, a perceived threat, that is discontinuation of exclusive breastfeeding as a result of insufficient production of breastmilk and breastmilk not being enough to satisfy the child leads to discontinuation of exclusive breastfeeding practice. In addition, other threats such as inconvenience, mothers’ weight, breast sag and dizziness were identified as challenges associated with exclusive breastfeeding. Furthermore, external factors such as friends, relatives and health workers served as cues to action that encouraged mothers to continue practicing exclusive breastfeeding. Information from health professionals and advertisement also support the practice of exclusive breastfeeding.

## Conclusion

This study sought to examine the experience of exclusive and non-exclusive breastfeeding mothers and health workers in Ghana. The findings revealed different levels of experiences that affect and shape the practice of exclusive breastfeeding. The decision to practice exclusive breastfeeding and the challenges of exclusive breastfeeding emanates from mothers’ personal experiences and institutional interactions. Based on the findings of the study, it is recommended that mothers who face challenges in practicing exclusive breastfeeding should be counselled and monitored to overcome these challenges. In addition, there is the need to increase the duration of maternity leave from the current three months to six months. There should also be frequent interaction between health workers and mothers to discuss breastfeeding experiences of mothers. Lastly, grandmothers and mothers-in-law should be educated on the benefits of exclusive breastfeeding so they can encourage breastfeeding mothers to practice exclusive breastfeeding.

## Supplementary Information

**Additional file 1. **

**Additional file 2. **

**Additional file 3. **

## Data Availability

The dataset used for the study is available and can be requested from the corresponding author on reasonable request.
