# Supplementary material for: Enablers and inhibitors of exclusive breastfeeding: perspectives from mothers and health workers in Accra, Ghana
Source: Int Breastfeed J. 2022 Mar 21;17:21. doi: 10.1186/s13006-022-00462-z (PMC8935745; doi:10.1186/s13006-022-00462-z)
Supplement: Supplementary file 2 — Additional file 2. [file 13006_2022_462_MOESM2_ESM.docx]

**Interview guide for Non-exclusive breastfeeding mothers**

**Section 1: Demographics**

1.1 What is your highest level of education?

1.2 What is your ethnic affiliation?

1.3 What is your religious affiliation?

Specify your denomination if a Christian?

1.4 What is your marital status?

1.5 What is your employment status? If yes, indicate what you do?

1.6 How many children do you have?

1.7 How old are you?

1.8 What is your age when you first gave birth or age for your last child?

1.9 Locality of residence?

1.10 Sex of child?

1.11 Mode of delivery?

1.12 How old is your child?

**Section 2: Child characteristics**

2.1 What was the birth size of your child when he/she was born?

2.2 Were you satisfied or not satisfied with the size of your child when he/she was born?

Probe: Reason for been satisfied or not satisfied

2.3 What was the birth weight of your child? (Check weighing card book to record)

2.4 Were you informed about the birth weight of the child after delivery?

2.5 Did you consider the birth size or birth weight to be more important to you?

**Section 3: Decision to exclusively breastfeed**

3.1 Did you plan not to exclusively breastfeed your child after delivery?

Probe: Reason for intention not to exclusively breastfeed

3.2 What informed your decision NOT to practice exclusive breastfeeding?

Probe: Birth weight and birth size

Probe: Were you told at the hospital to practice exclusive breastfeeding? if yes, by who?

Probe: Were you taken through nutrition lessons during antenatal clinics and after delivery

breastfeeding management? If yes, what lesson

Probe: Partner support, in-law support, economic reasons

**Section 4: Challenges of exclusive breastfeeding**

4.1 Why you are not practicing exclusive breastfeeding?

Probe: Challenges from the child

Challenges from the mother

Social, religion, work, environment, family, friends

4.2 Are there cultural issues promoting the practice of exclusive breastfeeding?

Probe: for local beliefs

4.3 Are there cultural issues inhibiting the practice of exclusive breastfeeding?

Probe: for local beliefs
