# Supplementary material for: Enablers and inhibitors of exclusive breastfeeding: perspectives from mothers and health workers in Accra, Ghana
Source: Int Breastfeed J. 2022 Mar 21;17:21. doi: 10.1186/s13006-022-00462-z (PMC8935745; doi:10.1186/s13006-022-00462-z)
Supplement: Supplementary file 3 — Additional file 3. [file 13006_2022_462_MOESM3_ESM.docx]

**In-depth Interview guide for health workers**

1. Can you please tell me something yourself?
2. Can you please provide a brief description of your work?
3. Do you inform mothers about the birth weight of their child after delivery?
4. What is your view on exclusive breastfeeding?

Probe for policy on EBF

1. Are mothers taken through nutrition lessons during antenatal and postnatal care?
2. What do you tell mothers with low birth weight, normal birth weight and high birth weight about exclusive breastfeeding?
3. Do you inform mothers to practice exclusive breastfeeding?
4. Why do you think mothers’ practice exclusive breastfeeding?
5. What are the reasons why mothers don’t practice exclusive breastfeeding?
6. What are the challenges you faced in telling mothers to practice exclusive breastfeeding?

Probe: Misconceptions relating to EBF

1. What are the cultural issues promoting the practice of exclusive breastfeeding?
2. 1What are the cultural issues inhibiting exclusive breastfeeding?
3. How can exclusive breastfeeding be improved?
